# The impact of provider-initiated (opt-out) HIV testing and counseling of patients with sexually transmitted infection in Cape Town, South Africa: a controlled trial

**DOI:** 10.1186/1748-5908-5-8

**Published:** 2010-01-30

**Authors:** Natalie Leon, Pren Naidoo, Catherine Mathews, Simon Lewin, Carl Lombard

**Affiliations:** 1 Health Systems Research Unit, Medical Research Council of South Africa (MRC), Cape Town, South Africa; 2 Independent Public Health Consultant, Cape Town, South Africa; 3 School of Public Health and Family Medicine, University of Cape Town (UCT), Cape Town, South Africa; 4 Preventive and International Health Care Unit, Norwegian Knowledge Centre for the Health Services, Oslo, Norway; 5 Biostatistics Unit, Health Systems Research Unit, Medical Research Council of South Africa (MRC), Cape Town, South Africa

## Abstract

**Background:**

The effectiveness of provider-initiated HIV testing and counseling (PITC) for patients with sexually transmitted infection (STI) in resource-constrained settings are of particular concern for high HIV prevalence countries like South Africa. This study evaluated whether the PITC approach increased HIV testing amongst patients with a new episode of sexually transmitted infection, as compared to standard voluntary counseling and testing (VCT) at the primary care level in South Africa, a high prevalence and low resource setting.

**Methods:**

The design was a pragmatic cluster-controlled trial with seven intervention and 14 control clinics in Cape Town. Nurses in intervention clinics integrated PITC into standard HIV care with few additional resources, whilst lay counselors continued with the VCT approach in control clinics. Routine data were collected for a six-month period following the intervention in 2007, on new STI patients who were offered and who accepted HIV testing. The main outcome measure was the proportion of new STI patients tested for HIV, with secondary outcomes being the proportions who were offered and who declined the HIV test.

**Results:**

A significantly higher proportion of new STI patients in the intervention group tested for HIV as compared to the control group with (56.4% intervention versus 42.6% control, p = 0.037). This increase was achieved despite a significantly higher proportion intervention group declining testing when offered (26.7% intervention versus 13.5% control, p = 0.0086). Patients were more likely to be offered HIV testing in intervention clinics, where providers offered the HIV test to 76.8% of new STI patients versus 50.9% in the control group (p = 0.0029). There was significantly less variation in the main outcomes across the intervention clinics, suggesting that the intervention also facilitated more consistent performance.

**Conclusions:**

PITC was successful in three ways: it increased the proportion of new STI patients tested for HIV; it increased the proportion of new STI patients offered HIV testing; and it delivered more consistent performance across clinics. Recommendations are made for increasing the impact and feasibility of PITC in high HIV prevalence and resource-constrained settings. These include more flexible use of clinical and lay staff, and combining PITC with VCT and other community-based approaches to HIV testing.

**Trial registration:**

Controlled trial ISRCTN93692532

## Background

More than two decades into the AIDS epidemic, the majority of people living with HIV are unaware of their status. In 2005, surveys showed that in Sub-Saharan Africa, the epicentre of the epidemic, only 8% to 25% of people living with HIV knew their sero-status [1]. Low testing rates have been linked to a reliance on Voluntary Counseling and Testing (VCT) as the sole approach to HIV testing [2]. Barriers to VCT include low perceived risk for HIV infection, negative perceptions of testing services, lengthy pre- and post-test counseling and shortages of counselors [2-5].

Provider-initiated HIV testing and counseling (PITC), (sometimes referred to as 'routine offer of testing' or 'opt-out testing') is a streamlined model promoted by the World Health Organization (WHO) and The Joint United Nations Program on HIV/AIDS (UNAIDS) to increase the opportunities for diagnosing HIV in health facilities, especially in high prevalence countries [6]. In the PITC approach, all patients are offered HIV testing routinely by the clinician as part of standard medical care, regardless of their presenting complaint. Testing remains voluntary and the patient is given the option to 'opt-out' of testing [6]. The aim of PITC is to decrease barriers to testing in order to increase testing rates and thereby facilitate earlier access to HIV treatment and prevention. Early access to treatment has been shown to reduce morbidity and mortality [7,8]. There is also evidence that knowledge of HIV-positive status can reduce risk behaviour and transmission rates, especially among sero-discordant couples [9-11].

Evidence from both low- and high-income countries, mainly from antenatal and tuberculosis (TB) settings, indicates that the direct offer of HIV testing by health providers can result in significant improvements in test uptake. Effect sizes range from as low as 5% [12] to as high as 50% [13], while baseline testing rates can vary from 6% to as high as 75% depending on setting [2,6,7,14]. There are early indications that PITC may work in Sub-Saharan Africa, but the evidence is limited to a few studies among antenatal and TB patients, and one with patients with sexually transmitted infection (STI). For example, in Botswana, a significant increase was reported in antenatal patients who knew their status (47% to 78%) after introducing PITC [15]. In South Africa, a cluster randomised trial with newly registered TB patients showed an increase in testing rates from 6.5% to 20.2% (p = 0.009) [16], and a quasi-experimental study among hospital outpatients showed an increase in case detection from an average of eight to 39 HIV cases per week (p < 0.0001) [17].

Patients with STI, another important high-risk group, have been less well-studied, particularly in high prevalence settings, perhaps because of the relative newness of the PITC approach. Given the strong association between STIs and the risk of acquiring and transmitting HIV [8,18,19], improving HIV detection and treatment among STI patients is a pressing issue. Studies in high-income countries have found high rates of missed opportunities for HIV testing among STI patients, with up to 70% of HIV infections remaining unrecognized [18,20-22], and a study in Malawi indicated that an alarming number of acute HIV infections are missed in sexually transmitted disease (STD) clinics [23]. It cannot be assumed, however, that the PITC success with antenatal and TB patients will apply to STI patients because there are other factors that may act as incentives to test among TB and antenatal patients. The acute, non-recurring nature of STI services also means there are limited opportunities to offer HIV testing and thus to increase testing rates. Furthermore, there are important concerns about the feasibility and ethics of PITC in resource-constrained settings (many of which are also high HIV prevalence areas), because it may be difficult to add PITC effectively to the clinical workload in under-resourced health services [4,24].

The evidence for PITC with STI patients is limited to a few quasi-experimental studies in the UK and the Netherlands [20,21,25,26], and one controlled trial in Sub-Saharan Africa [27]. The European trials report significant increases in HIV testing for STI patients, although a recent UK study with low-risk STI patients showed that there was a significant increase in the offer of testing, not in the proportion of patients who accepted testing [28]. The one Sub-Saharan African PITC study with STI patients was a controlled trial in Botswana that showed a significant increase in HIV testing for STI patients (33% in intervention clinics as compared to 14% in control clinics, p < 0.001) [27]. However, PITC was one of four clinical interventions that made up the training intervention in this study, making it difficult to isolate the impact of PITC alone. No randomised control trials on PITC for STI patients have been reported.

The close association between HIV and STI, the high rates of missed opportunities for testing STI patients, and limited evidence on the effectiveness of PITC for STI patients in high prevalence and resource-constrained settings are of particular concern for high prevalence countries like South Africa. In April 2006, the Cape Town health authorities undertook a trial to evaluate the impact of PITC on the testing rates of STI patients, compared to the standard VCT approach at primary care level. In order to evaluate PITC in as realistic a context as possible, the intervention was implemented by existing health managers and with few additional research or clinical resources. This study therefore addresses a key gap in evidence regarding the effectiveness of PITC for STI patients in high prevalence and resource-constrained settings.

## Methods

### Study setting

The study was performed in the public sector, primary healthcare services in Cape Town, South Africa. STI services are free of charge and are delivered mostly by trained nurse practitioners. VCT is the standard approach and is provided by trained lay health counselors. At the start of the intervention in 2006, the total new STI caseload for Cape Town was more than 5,000 patients per month, with an estimated 30% HIV testing rate. Although Cape Town has amongst the lowest HIV prevalence in the country, the prevalence varies dramatically between sub-districts. In 2005, the average HIV prevalence for pregnant women was 12.7% [29], whilst some of the poorest sub-districts had rates of over 30% which are amongst the highest in the country [30].

The health authority gave permission for the evaluation, and ethical approval was obtained from the University of Cape Town Research Ethics Committee. A confidentiality agreement was signed between the health authorities and the research team to ensure additional protection of patient information.

### Study design

The design was a pragmatic cluster non-randomised controlled trial, in which seven clinics were selected to receive the intervention, and 14 clinics served as control clinics. The trial aimed to test the effect of the intervention under normal operational and management conditions. Randomization was not feasible because the intervention clinics were selected before the evaluation was planned. The assessment of the intervention was facilitated by the health management team, which also made it impractical to blind the implementers and the assessors.

The primary outcome was the HIV testing rate amongst new STI clients. Secondary outcomes were the proportion of STI clients offered HIV testing (irrespective of whether they accepted or not) and the proportion that declined HIV testing, once offered. We also describe the sample in terms of the participant's gender, age and proportion diagnosed with HIV.

### Study population and sampling

A pool of 24 clinics was identified by the health services as eligible to participate, based on criteria of geographical representation (one clinic from each of the health sub-districts), nurse-patient ratio, and a minimum STI caseload of 30 new STI patients per month. A project steering committee, in consultation with local district managers, selected one representative clinic from each district. Nine intervention sites were selected initially, but two clinics declined participation shortly before implementation, citing operational difficulties. One control clinic opted out, citing similar reasons. The remaining fourteen eligible clinics became the comparison group. There was no matching of clinics, but statistical comparison of clinics at baseline was conducted using general routine administrative data that were collected retrospectively.

A sample size validation was done, given that the intervention arm would consist of seven intervention clinics. With this restriction, the study would be able to show an increase of 20% in testing rate from an estimated baseline of 30%, with 80% power, at a 5% significance level and using an intra-class correlation coefficient (ICC) of 0.08 and a cluster size of 90. In a recent review of ICCs in 188 health systems research studies, the median ICC used was 0.051 (IQR 0.011 to 0.094). The ICC of 0.08 in this study is closer to the conservative end of this range [31]. We doubled the control group to 14 clinics to increase the power of the study. Based on available data, we assumed a cluster size of 90 new STI patients per quarter per clinic.

### The PITC intervention

This intervention is an adapted version of the 'ACTS' approach which includes four brief steps: assess, get consent, test, and provide supportive services [32]. In this PITC intervention, the STI nurse offered HIV testing as a standard part of STI care for all STI clients, and the client had to decline or 'opt-out' of this testing. According to policy in South Africa, written consent was required (although the WHO guidelines for PITC allow for only verbal consent). Abbreviated pre-test counseling consisted of informing patients that HIV is an STI and recommending that they test for HIV at this consultation. If they agreed, the nurse would do a brief test readiness assessment, obtain written informed consent, and perform the rapid test along with other routine blood tests such as those for syphilis.

By using clinical staff to deliver the intervention as an integrated part of the STI consultation, the intervention departed significantly from the standard approach in South Africa where VCT is predominantly provided by lay counselors. The shift required a reconfiguration of roles for nurses and lay counselors, with nurses taking on some of the tasks previously performed by lay counselors. It was anticipated that more patients would be tested because the clinical care setting provided the opportunity to offer HIV testing to all patients, and the setting may also increase patient willingness to test. Because this was a demonstration project to evaluate also the feasibility and acceptability of using clinical staff for HIV screening (instead of lay workers), the intervention needed to address concerns about the possible overloading of nurses. To save nurse time, nurses were allowed to refer the patient to the clinic lay counselor for the test result and post-test counseling once they completed the STI consult and HIV testing.

STI nurses from each of the intervention sites received a two-day training course on PITC and how to implement it. Lay counselors also received training to emphasize follow-up support for HIV positive clients. The implementation was standardized in terms of the main components of integrated nurse-initiated testing, but clinics were allowed to adapt their patient flow arrangements to suit their clinic. No additional nursing resources were provided to implement the intervention at clinic level, but a part-time project manager from within the health service allocated 30% of her time to oversee the implementation, supervision, and monitoring of the intervention. Intervention clinics received quarterly feedback about their performance.

In control sites, the routine VCT approach continued. The VCT service is available at the same site as the STI service, and any person can access the VCT service on his or her own initiative or via medical referral. The majority of patients using the VCT service would be required to initiate testing themselves, whilst a few would be medically referred if they had HIV-related symptoms or if considered high risk. In addition to patient barriers to access such as low perceived risk and poor perception of testing services, the lengthy pre-and post-test counseling and shortages of counselors are potential barriers to VCT access in these settings [2-5].

In the VCT service, lay counselors provided pre- and post-test counseling that lasted an average of 45 minutes [33]. This excluded waiting time which can be considerable in public sector clinics. Due to limits in the scope of practice of lay workers, a nurse had to perform the rapid and confirmatory tests, which added to the duration of VCT and also fragmented the VCT service. In this study, the pre-test counseling session offered by the lay counselor in the VCT approach and the abbreviated pre-test counseling offered by the nurse in the PITC intervention are both captured by the outcome 'offered HIV testing'.

### Data collection and analysis

#### Data sources and data management

Baseline data for 2005, the year prior to the intervention, were collated for intervention and control clinics to determine how the two groups compared on clinic demographics and health service delivery for STI and HIV testing services. The baseline data summary (Table 1) describes the intervention and control groups according to characteristics that may have influence on the outcomes. Although TB treatment outcomes are not directly related to STI and HIV service outcomes, it was included, as this indicator does reflect how well clinics function in a priority service area.

**Table 1:** Baseline comparison of intervention and control clinic demographics and service profile

Caseload	Intervention clinics (N = 7)	Control clinics (N = 14)	P value
1. Total caseload: annual number of patients treated in 2005	504,679	822,395	0.40

2. Adult caseload: number of patients who were 5 yrs and older	334,758	600,142	0.66

**STI services**			

3. STI-new: number of patients treated who presented with a new episode of STI	8,466	12,377	0.26

4. STI load as a proportion of total adult caseload	3%	2%	0.17

**HIV testing services**			

5. VCT Total: number of patients who received voluntary counseling and testing (VCT)	13,275	19,426	0.33

6. VCT load as a proportion of total adult caseload	4%	2%	0.94

7. Proportion of VCT patients who were female	56%	54%	0.33

8. HIV test acceptance, VCT: proportion of VCT patients who were tested for HIV	93%	85%	0.03*

9. HIV positive rate amongst patients who tested for HIV	29%	28%	0.82

10. Lay Counsellor workload: the average number of VCT patients counseled per lay counsellor per day	4.3 patients	4.7 patients	0.59

**TB treatment outcomes**			

11. VCT for TB: proportion of New Smear Positive TB patients who received VCT	79%	77%	0.63

12. TB success rate: proportion of New Smear Positive TB patients who were successfully treated (combined TB cure and TB completion rates)	79%	77%	0.97

Annual data for 2005

Data sources for variables numbered: 1 to 4 = Routine Monthly Report (RMR); 5, 7, 8, 9, and 11 = Voluntary Counseling and Testing quarterly reports; 10 = Quarterly lay counsellor statistics report, 2005; 12 = TB quarterly reports.

The intervention was evaluated approximately one year after the start of implementation, using six months of routine data from January to June 2007. The routine monthly report (RMR) collated by the health services provided data on the numbers of new STI patients. HIV testing data were collated from the counseling and testing (CT) register that included the patient demographics of new STI patients offered HIV testing, whether they accepted the test, and the HIV test result. The CT register recorded the type of patients, including antenatal and TB patients, and was adapted to record STI patients as a separate category.

Data from the CT register was entered into an Excel database. Data were linked to patient identifiers, and for this part of the study we recorded patient record numbers, but not patient names. Data quality checks were done in stages. This involved checking the RMR data, paper copies of the CT registers, and the electronic data set for completeness and accuracy. Problems such as incomplete and inaccurate CT registers (including missing patient identifiers and missing or inconsistent testing information) were queried and corrected by clinic staff through checking patient records. The 10% sample check at the end identified a particular clinic where there were anomalies in the data (mainly for gender and age) and this was corrected. This did not require a rechecking and re-entry of the whole data set.

### Statistical methods

The proportion of patients offered testing, tested, and declined per clinic was calculated. For the variables 'HIV tested', 'not tested' and 'offered HIV testing' the denominator was the total number of new STI patients treated in these clinics. For variable 'declined HIV testing' the denominator was the total number of new STI patients who were offered HIV testing, in other words, a subgroup of the total new STI population. These proportions were utilized as clinic level outcomes and used to compare the two groups. This clinic level analysis accounts for the clustering within clinics [34].

The statistical comparison was done using STATA 10. A two-sample t-test was used for the comparison of the study outcome and baseline data between the intervention and control group. We checked the normality assumption of the t-test. A formal test for the equality of variances in the two groups was done using the F-test. The appropriate t-test was subsequently performed based on the homogeneity status of the variances. Multinomial logistic regression analysis with adjustment for clustering was done on the composite testing outcome ('tested', 'not tested 'and 'not offered') to confirm the results of the clinic level analysis. Only the clinics that were operationally able to implement the protocol and provide outcome data to the trial were included in the analysis because we wanted to know the effectiveness of the PITC intervention in clinics that actually implemented the intervention. The statistical adjustment for baseline differences is not reported and this is considered in the results and in the study limitations.

## Results

**Figure 1:**
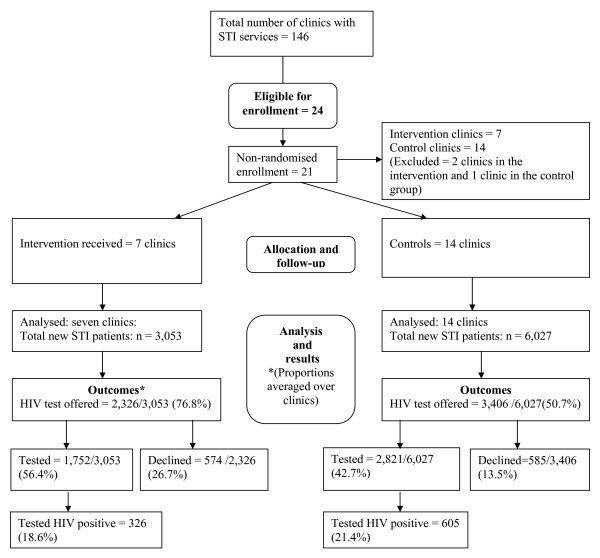
**Flow chart for PITC controlled trial**.

The flow chart in Figure 1 provides a profile of the enrolment, analysis, and results of the trial. It includes total raw numbers across all clinics along with the appropriate denominator for each outcome. Note, however, that the proportions in parentheses are averaged over the clinics to account for clustering and are thus not the same as the proportions for the raw numbers presented.

### Baseline comparison

Baseline data on clinic demographics and service delivery from the start of the intervention in 2005 are provided in Table 1. There were no significant differences between the intervention and control groups for either their STI and HIV testing service delivery characteristics or their TB treatment outcomes, except for the HIV test acceptance, VCT variable (p = 0.03).

### Proportion tested, offered testing and declined

**Figure 2:**
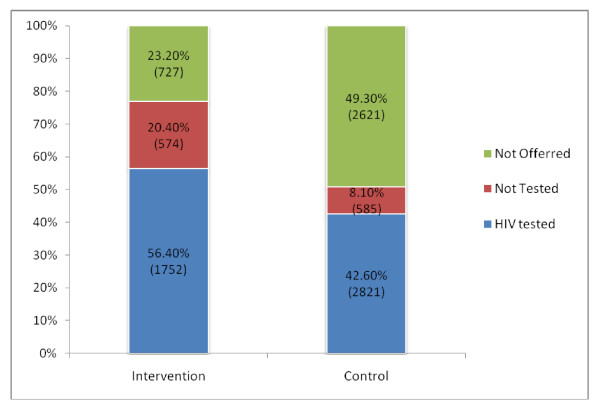
**'HIV tested', 'Not tested' and 'Not offered' in intervention and control groups**.

Clinic level raw data and results are shown in Table 2, with the proportions for the main outcomes averaged over clinics. A total of 9,080 new STI patients were treated during the study period; 3,053 in the intervention and 6,027 in the control group. The main results are shown in Figure 2 and the corresponding Table 3. The two bars in Figure 2 represent 100% of the new STI patients seen in the period of January to June 2007 in both intervention and control clinics. A significantly greater proportion of new STI patients were tested for HIV in the intervention compared to the control group (56.4% intervention versus 42.6% control, p = 0.037). The increase of 13.8% in test uptake with the new intervention means that for every seven new STI patients treated, one additional HIV test was achieved (NNT = 7.3).

**Table 2:** Comparison of outcomes for the intervention and controls per clinic ('offered HIV testing', 'HIV tested', 'not tested for HIV' and 'declined HIV testing').

	STI Total	Offered HIV testing (%) (Total offered/Total STI)	HIV tested (%) (Total tested/Total STI)	Not tested for HIV (%) (Total declined/Total STI)	Declined HIV testing (%) (Total declined/Total offered)
**Intervention**					

1	451	363 (80.5)	256 (56.8)	107 (23.7)	107 (29.5)

2	520	400 (76.9)	306 (58.8)	94 (18.1)	94 (23.5)

3	412	346 (84.0)	236 (57.3)	110 (26.7)	110 (31.8)

4	850	572 (67.3)	492 (57.9)	80 (9.4)	80 (14.0)

5	425	338 (79.5)	228 (53.6)	110 (25.9)	110 (32.5)

6	249	215 (86.3)	177 (71.1)	38 (15.3)	38 (17.7)

7	146	92 (63.0)	57 (39.0)	35 (24.0)	35 (38.0)

Total*	**3053**	**2326 (76.8)**	**1752 (56.4)**	**574 (20.4)**	**574 (26.7)**

**Control**					

8	421	87 (20.7)	86 (20.4)	1 (0.2)	1 (1.1)

9	174	119 (68.4)	105 (60.3)	14 (8.0)	14 (11.8)

10	388	129 (33.2)	120 (30.9)	9 (2.3)	9 (7.0)

11	166	51 (30.7)	47 (28.3)	4 (2.4%)	4 (7.8)

12	593	197 (33.2)	165 (27.8)	32 (5.4)	32 (16.2)

13	789	669 (84.8)	636 (80.6)	33 (4.2)	33 (4.9)

14	837	684 (81.7)	534 (63.8)	150 (17.9)	150 (21.9)

15	626	333 (53.2)	221 (35.3)	112 (17.9)	112(33.6)

16	320	25 (7.8)	24 (7.5)	1 (0.3)	1 (4.0)

17	373	135 (36.2)	103 (27.6)	32 (8.6)	32 (23.7)

18	164	27 (16.5)	27 (16.5)	0 (0.0)	0 (0)

19	285	211 (74.0)	182 (63.9)	29 (10.2)	29 (13.7)

20	576	449 (78.0)	327 (56.8)	122 (21.2)	122 (27.2)

21	315	290 (92.1)	244 (77.5)	46 (14.6)	46 (15.9)

Total*	**6027**	**3406 (50.7)**	**2821 (42.7)**	**585 (8.10)**	**585 (13.5)**

*Note that all the proportions in the 'Total' columns are averaged over clinics to account for clustering

**Table 3:** Proportion of new STI patients who were 'HIV tested', 'offered HIV testing' and 'declined HIV testing' in the intervention and control groups.

	Intervention N = 7	Control N = 14	P value *(p < 0.05)
**HIV tested** **(% of total STI patients tested for HIV)**	56.4% (CI: 49.4-63.2)	42.7% (CI: 31.4-53.8)	0.037*

**Clinic range**	39 to 71.1%	7.5 to 80.6%	0.033*

**Offered HIV testing** **(% of total STI patients offered HIV testing)**	76.8% (CI: 68.8 to 84.0)	50.7% (CI: 34.3 to 72)	0.0029*

**Clinic range**	63% to 84%%	7.8% to 92.1%	0.0076*

Declined HIV testing** (% of STI patients *offered*, but declined testing)	26.7% (CI:18.70 to 4.7)	13.5% (CI: 7.6 to 19.4)	0.0086*

**Clinic range**	Range: 14 to 38%	Range: 0 to 33%	0.71

**Two-sided t-test since no direction was hypothesized.

Patients were also more likely to be offered HIV testing in the intervention clinics: intervention clinics offered the HIV test to 2,326 (76.8%) of new STI patients compared with 3,406 (50.7%) in the control group (p = 0.0029). In Figure 2, the proportion of patients offered testing is made up of a combination of the blue ('HIV tested') and red ('not tested') sections of the graph. The 'not tested' variable represents the patients in the CT register who were offered testing but who declined this. The proportion of patients who declined testing ('not tested') in the figure is calculated as a proportion of all new STI patients.

Another way to measure those who declined testing is as a proportion of those offered testing. This alternative calculation represents test refusal within the context of those offered the test rather than all new STI patients and is reflected by the variable 'declined HIV testing' in Figure 1, Table 2 and Table 3. Of those patients offered HIV testing, a significantly greater proportion (26.7%) declined testing in the intervention group than in the control group (13.5%), p = 0.0086.

As mentioned earlier, the adjusted baseline analysis is not used. The inverse association observed between the baseline variable of a broader population and the much narrower study population may lead to a distorted estimate of the intervention effect. The unadjusted results reported in the manuscript is a more conservative estimate of the intervention effect when compared to the adjusted estimate given in this analysis (13.8% compared to 22.2%). We feel that the unadjusted result is a more reasonable result with respect to the health system setting in which this was done.

From the multinomial regression (adjusted for clustering) on the composite testing outcome (tested, not tested, not offered) the odds ratio for being tested is 2.24 (95% CI: 1.12 to 4.46), p = 0.022 for the intervention group compared to the control group with the reference category being 'not offered'. This result confirms the outcome specific comparison reported in Figure 2 and Table 3.

### Variance in clinic performance

Although not a pre-specified outcome measure, the data suggested wide variability in range for outcomes measured in the clinics, and this was compared statistically using the F test. As shown in Table 3, there was significantly less variation in clinic performance in the intervention group for both 'HIV tested' (intervention: 39% to 71%; control: 8% to 81%; p = 0.033) and 'HIV test offered' rates (intervention: 63% to 84% control: 7.8% to 92%; p = 0.0076). Of note is that two control clinics (clinic number 13 and 21 in Table 2) achieved higher testing rates than the intervention sites (80.6% and 77.5%, respectively, as compared to the highest rates of 71,1% in the interventions clinics).

### Gender profile, age, and HIV diagnosis of study participants

There were no significant differences in the male to female ratio between the intervention and control groups on any of the outcomes examined (Table 4). Of those who tested, a similar proportion was male (43%) in both groups. There was no significant difference between intervention and control clinics in the proportion of patients who tested HIV positive (18,6% in the intervention and 21,4% in the control group, p = 0.147).

**Table 4:** Gender, age, and HIV diagnosis for study participants in the intervention and control groups.

	Intervention	Control
**Proportion tested who were male**	42.8% (n = 751)	42.5% (n = 1200)

**Proportion offered testing who were male**	43.9% (n = 1,012)	43.7% (n = 1490)

**Proportion who declined testing who were male**	47% (n = 270)	49.6% (n = 290)

**Mean age of patients tested for HIV**	26 yrs	28 yrs

**HIV positive diagnosis for tested patients**	18.6% (n = 326)	21.4% (n = 605)

## Discussion

A streamlined HIV testing protocol that is integrated into STI care and delivered by nurses in busy primary healthcare clinics in Cape Town appears to be effective and feasible. PITC significantly increased the proportion of new STI patients who tested for HIV as well as the proportion of patients offered testing. There was also significantly less variation in the main outcomes across the intervention clinics, suggesting that the intervention facilitated more consistent performance.

The increase in testing rate was achieved even though only 76% of all STI patients in the intervention clinics were offered testing. One area for increasing the effectiveness of the PITC intervention is in increasing the coverage of the offer of testing to 100% of new STI patients. More than one-quarter of patients offered testing in the intervention group declined to test (twice the proportion in the control group), a result that is not favourable to the intervention. The result is understandable given that patients did not self-initiate testing, as is the case for VCT. Nevertheless, reducing the proportion who decline testing represents another area where the impact of the PITC intervention could be improved substantially. This could be done by training staff to use more effective motivation strategies to encourage patients to test, provided that this does not compromise informed consent from patients. The high test refusal rate found could be considered indirect evidence that patients were able to exercise their right to decline testing and is therefore an indirect marker of the ethical implementation of the PITC approach.

The absolute increase in HIV testing rate of nearly 14% is smaller than the difference of 20% anticipated prior to the study, though it is similar to increases reported in prior studies in South Africa (13% increase for TB patients) [16] and Botswana (19% increase for STI patients) [27]. This study may have underestimated the impact of the PITC intervention as two practice shifts could have narrowed the difference between control and intervention clinic outcomes in the study setting. First, following revisions to the CT register, all clinics were able to record the testing of new STI patients. Second, midway through the intervention period, the health authority set a 50% HIV testing target for new STI patients across the city. Both of these changes might have incentivised control clinics to improve their performance. However, this suggestion remains speculative due to the lack of baseline testing rates for STI patients.

The findings have important operational implications beyond the testing rate itself and relating to the management of staff resources. In this study, STI nurses integrated HIV screening into their STI consultations, taking on a task previously performed by lay health workers. This is unlike the task shifting recommended by WHO, where tasks are referred down to less skilled workers to free up clinical time for treatment and care of HIV (Type III task shifting) [35,36]. However, it provides a rational way of utilizing existing staff resources where there is limited capacity to recruit more lay workers. The change in nursing roles was achieved with relatively short training and by extending slightly their consultation time (by approximately five to seven minutes on average). Nevertheless, nurses were not able to implement the intervention in full with all their STI clients and tended to drop the offer of testing during busy periods. For nurses to increase the offer of testing from 76%, as measured in this study, to 100% of new STI patients, they may require even more streamlined protocols for the integration of HIV screening into clinical practice.

Where nursing resources are very limited, it may be less feasible to use only clinical staff for PITC, and lay providers could be used in such settings [37]. In neighbouring Botswana and Lesotho, the two countries at the forefront of PITC in Africa, logistical and resource constraints to implementing PITC have already emerged, and there are associated concerns about compromising patient informed consent [4]. It is possible that such problems may increase as the intervention is implemented to scale, and supervision and support are reduced.

Successful scaling up of this PITC intervention in high prevalence and resource-constrained settings will require adaptation to the local context, including a flexible approach to the utilisation of clinical and lay staff. Retraining of nurses and lay counselors may also be necessary so as to streamline and integrate HIV testing more effectively. HIV testing rates could be increased further through expanding PITC to other categories of clinic patients; by decreasing the test refusal rate; and possibly by reducing the requirement for written consent. It is interesting to note that two control clinics achieved higher testing rates with the VCT approach than the intervention sites. Further enquiry suggested that their success was due to the close supervision and monitoring of the VCT staff team, factors that are also likely to be important for the successful up-scaling of PITC.

The findings of this study contributed to the decision to roll-out a more streamlined version of PITC to several sub-districts in Cape Town in 2008. The health authorities are now formulating policy to scale up the intervention to the provincial level.

### Generalisability

As a pragmatic trial, this intervention was implemented using existing management and clinical staff resources and some internal project management support. This situation, where the service managers initiate and run the intervention and where there is little additional resources added, is an ideal context for an effectiveness evaluation as it reflects the reality of implementation in resource-constrained settings [38,39]. The estimated baseline testing rate for STIs of 30% in this study may be higher than in other low resource settings, and the staff configurations for HIV service delivery may also differ. These factors could influence the size of the impact of the intervention in other settings.

### Limitations of the study

The lack of randomization of clinics to intervention and control groups introduces the risk of bias for the study outcomes. While the baseline comparison showed few differences between intervention and control sites, we cannot exclude other unknown sources of selection bias. Also, we were not able to obtain baseline measures for the main outcome. The one baseline testing variable (HIV test acceptance, VCT) that was significantly different across intervention and control sites was inappropriate as a measure of baseline differences for the following reasons: this variable is not strictly comparable to the main outcome in this study because it refers to the proportion of general clinic patients who were tested as a proportion of those who received pre-test counseling by lay counselors in the VCT service; and it covers a different and much larger general patient target group (and not STI patients only) and refers to a different testing service (VCT), delivered by lay counselors and not clinicians. However, given the lack of baseline data on the main outcome, we felt that this 'HIV test acceptance, VCT' variable should be examined in the baseline comparison of the two groups, and that unadjusted outcome measures should be presented. In the absence of blinding, factors such as staff enthusiasm and increased monitoring could have had a modifying effect. As mentioned earlier, we cannot be certain about how the unforeseen management efforts to increase HIV testing rates, implemented in parallel to this intervention, may have affected the study results.

## Summary

To our knowledge, this is only the second study on the impact of the PITC approach for STI patients in a high prevalence African country (the other being in Botswana), and the first where the study design was able to isolate the effect of the PITC intervention. The study demonstrates that PITC, integrated into STI care and delivered by nurses in an urban, primary healthcare setting had three successes: it increased the coverage of HIV testing opportunities, it increased testing uptake, and it delivered more consistent performance across clinics.

PITC is recommended as an effective strategy for increasing the uptake of HIV testing in medical settings in high prevalence and resource-constrained countries such as South Africa. However, this study indicates that this approach alone may not deliver the large-scale increases in testing required in high prevalence settings. A multi-faceted strategy to expanding HIV testing and care may be needed. This should combine PITC with efforts to optimise existing VCT services. Creative options for community, home-based, and self testing for HIV should also be introduced and evaluated. Finally, efforts to increase testing rates should be matched by efforts to link newly diagnosed HIV positive patients to clinical care and prevention services because this is ultimately the aim of HIV testing.

## Competing interests

The authors declare that they have no financial conflict of interest. PN was the HIV manager in the health authority overseeing the implementation of the PITC intervention. She resigned from the health department midway through the evaluation, and continued as a research team member.

## Authors' contributions

Conception, design and acquisition of data: NL, PN, CM, SL and CL; analysis and interpretation of data: NL, PN, and CL; drafting the manuscript: NL; critical revisions for intellectual content: NL, CM, SL, PN and CL. All authors read and approved the final manuscript.
